# Somatic embryogenesis from mature sorghum seeds: An underutilized genome editing recipient system

**DOI:** 10.1016/j.heliyon.2023.e23638

**Published:** 2023-12-13

**Authors:** Han Wu, Kuangye Zhang, Jia Li, Jiaxu Wang, Yanqiu Wang, Junchi Yu, Ling Cong, Youhou Duan, Fulai Ke, Fei Zhang, Zhiqiang Liu, Feng Lu, Zhipeng Zhang, Jianqiu Zou, Kai Zhu

**Affiliations:** a Sorghum Research Institute, Liaoning Academy of Agricultural Sciences, Shenyang, China; b Key Laboratory of Agriculture Biotechnology, College of Biosciences and Biotechnology, Shenyang Agricultural University, Shenyang, China

**Keywords:** Somatic embryogenesis, Regeneration, Sorghum, Seeds, Genome editing, Recipient

## Abstract

Somatic embryogenesis is a process of cell totipotency in vitro, whereby an embryogenic cell develops from vegetative tissues rather than from zygotes after double fertilization. Sorghum is a recalcitrant crop in genetic transformation; previous recipient systems have usually been from immature zygotic embryos, which needed more time and labors to prepare. Here, an efficient 2,4-dichlorophenoxyacetic acid (2,4-D)-induced somatic embryogenesis system from mature sorghum seeds was introduced. 2,4-D can induce two types of calli from a plumular axis section. Low-concentration 2,4-D (e.g., 2 mg/L) induces white and loose non-embryogenic calli (type 1), while high-concentration 2,4-D (e.g., 8 mg/L) induces yellow and compact embryogenic calli (type 2), which can be clearly distinguished by Sudan red staining. Germinating seeds have a long 2-day window for SE induction. Somatic embryogenesis can be enhanced by HDAC inhibitor, trichostatin A (TSA), a histone deacetylase treatment, which shows more SE productivity and a bigger size. Importantly, this easily prepared protocol does not show obvious genotype dependency in sorghum hybrids. In this study, a high-concentration 2,4-D-induced SE system was established from mature sorghum seeds. This finding provides a technical option for the genome editing recipient in sorghum.

## 1 Introduction

Plant cells exhibit typical totipotency, with a capacity for regenerating entire plantlets either from a natural regeneration process after wounding or from artificial tissue culture experiments in vitro [1,2]. Somatic embryogenesis is a kind of regeneration whereby entire embryos develop from vegetative plant tissues rather than from a fertilized egg [3,4]. Various plant regeneration protocols are widely utilized in industrial crop breeding [5] and genome editing [6]. Technically, 2,4-D (2,4-dichlorphenoxyacetic acid) is widely applied as the chemical inducer in a vast majority of somatic embryogenesis protocols [[7], [8], [9], [10]]. As a member of the Auxin family, 2,4-D treatment has been shown to interact with other signaling pathways on a transcriptional and epigenetic level during somatic embryogenesis [11,12]. A zygotic embryo is an ideal explant to induce SE. The efficiency of somatic embryogenesis from IZE (immature zygotic embryos) is usually the highest, around ca. 75% [10], while that of mature embryos from seeds is relatively lower at about 20% in *Arabidopsis* [7,11]. In addition, some chemical enhancers, such as HDAC inhibitor trichostatin A (TSA) and 5-azacytidine AzaC, can be used to improve the efficiency of somatic embryogenesis. TSA can induce upregulation of the embryonic transcription factors (eg. LEC1or ABI3) to maintain the somatic embryo characteristic rather than vegetative development. Similar effects were also found in AzaC treatment, DNA demethylation can promote recipient cell reprogramming and embryogenesis initiation. In essence, enhancers and usually affect explants on a physiological [12] and epigenetic level [13,14], thereafter improving regeneration. The chemical enhancers can even be applied to boost embryogenesis from microspore [13,14].

Sorghum is a widely cultivated, worldwide crop that is recalcitrant to genetic transformation, restricting the application of its genome editing. An easily prepared recipient system is a prerequisite for transformation [14]. Successful regeneration systems of sorghum usually come from immature zygotic embryos [14,15] and immature inflorescence-induced calli [16]; even protoplasts [15] derived from it; and recently leaf fragments [17]. Nevertheless, a transformation protocol relying on immature zygotic embryos usually shows strong genotype-dependency. What is more, a continuous year-round supply of IZE as a recipient requires cost-prohibitive greenhouse infrastructure, which is a financial burden for most academic units. Therefore, an easily prepared recipient system would benefit genome editing in recalcitrant sorghum. Mature seeds are an ideal material for producing vigorous embryogenic calli [18,19], somatic embryos [9,11] and even protoplasts [20], in that they shorten the recipient preparation process. Technically, 2,4-D concentration was usually low (ca. 2.5 mg/L) [18,19] and in line with lower SE yield comparing with IZE. Higher concentration of 2,4-D seems be underutilized in mature sorghum seeds regeneration system.

In this study, an efficient somatic embryogenesis system from mature sorghum seeds was introduced. We show that relatively high-concentration 2,4-D can effectively induce a vigorous SE callus from germinating plumular axis cells. The somatic embryogenesis rate reaches ca. 60%. The quality and quantity of induced somatic embryo was significantly boosted from mature sorghum seeds. Our findings offer an easy option, which requires less handling for sorghum recipient preparation.

## 2 Materials and methods

### 2.1 Plant materials

Mature seeds of hybrid sorghum variety ‘Jin-za No. 12’ [18], ’Liao-za No.27’, ‘Liao-za No.36’, ‘Liao-za No.52’ and ‘Liao-tian No.3’ were harvested in the field after accurate hybridization, then packed and stored at 4 °C. All seeds sowed in one given experiment were harvested and prepared from the same batches. ‘Liao-za No.27’, ‘Liao-za No.36’, ‘Liao-za No.52’ and ‘Liao-tian No.3’ were breeded by Sorghum Research Institute (National Sorghum Improvement Center), Liaoning Academy of Agricultural Sciences, which were wildly cultivated in north China (ca. 45,000 ha).

### 2.2 Somatic embryo culture

The somatic embryogenesis protocol was modified from published reports [11,21] (Table 1). Mature sorghum seeds were surface sterilized with 70% ethanol for 30 min, followed by 2% bleach for 3 h and then washed with autoclaved ultra-pure water three times before being sowed onto 25 mL of MS-solidified medium in 9 cm Petri dishes (Murashige and Skoog macro and micro elements and vitamins (Duchefa)) [19], 3% (*w*/*v*) sucrose, pH 5.8). 2,4-D was dissolved with 2 mL sodium hydroxide then diluted with autoclaved ultra-pure water to make 2 mg/mL stocks (0.22 μm filtration sterilization) to make the 2,4-D gradient range from 0 to 20 mg/L when pouring dishes. We then applied 15 seeds per Petri dish. The seeds were placed in a growth chamber with a 16 h/8 h day/night cycle (100 μmol m^−2^ s^−1^) at 25 °C. The number of embryogenic calli (type 2) was counted after 4 weeks of culture. We performed 3 replicates with 45 seedlings/replicate per treatment.

**Table 1:** Medium of somatic embrogenesis and regeneration.

Developmental Stages	Medium [22]
Callus induction	MS medium, 2,4-D 8 mg/L, sucrose 30 g/L, pH5.8, agar 10 g/L.
Shoot elongation	MS medium, IAA and 6BA 0.5 mg/L, sucrose 30 g/L, pH5.8, agar 10 g/L.
Root induction	MS medium, 2.5 mg IBA, proline 0.7 g/L, inositol 0.1 g/L, 10 g/L PVP, pH5.8, agar 10 g/L.

### 2.3 Chemical enhancer

HDAC inhibitor, trichostatin A (TSA), a histone deacetylase treatment was used as a chemical enhancer of somatic embryogenesis. We diluted 100 mg TSA with DMSO and sterilized filtration (Millipore SLGP033RB, Shanghai, China) to make 10 mL stock and storage at −20 °C.

### 2.4 Microscopy

**Fig. 1:**
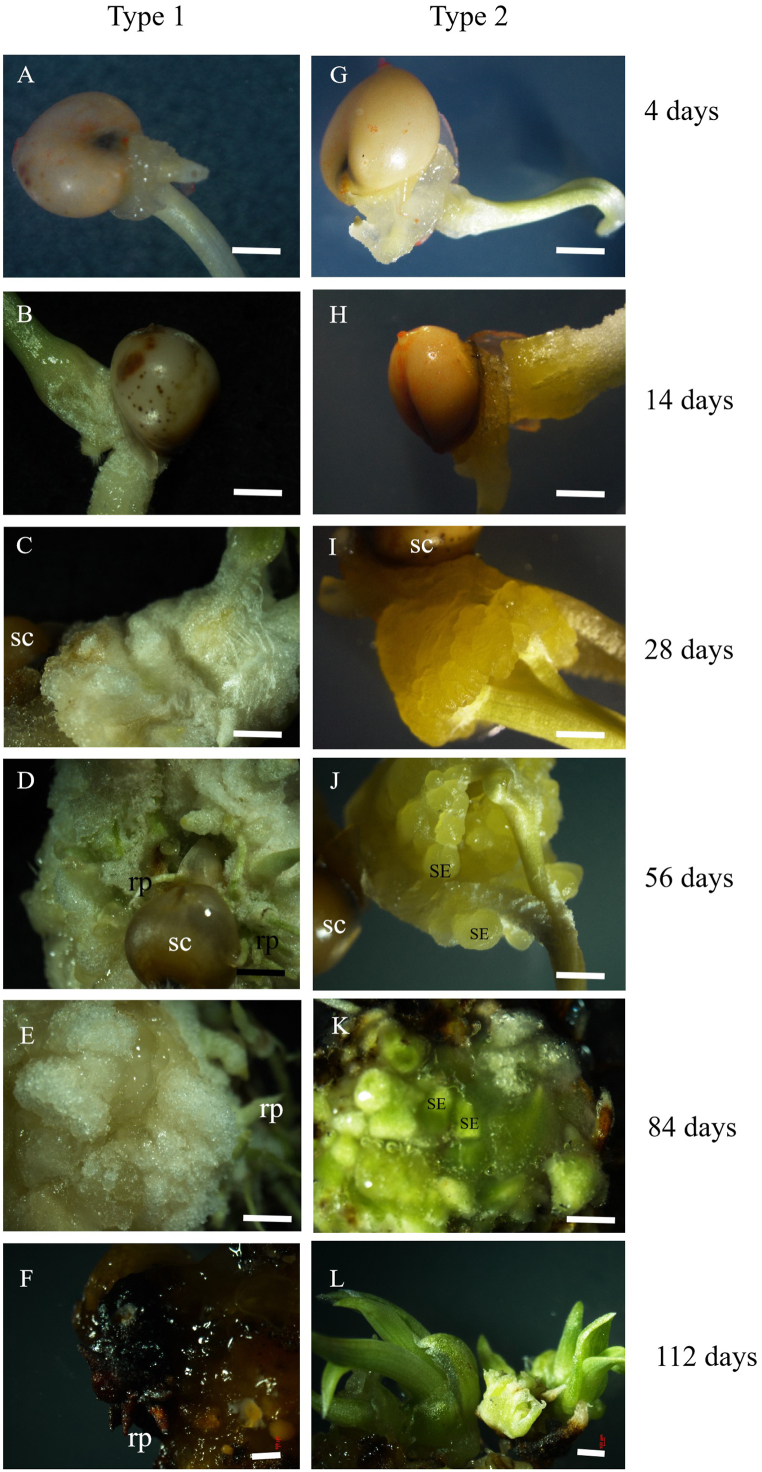
2,4-D induced two types of calli from mature sorghum seeds. Developmental process of the type 1 non-embryogenic callus (**A**–**F**); developmental process of the type 2 embryogenic callus (**G**–**L**). Magnified microscopic images: rp, root primordia; sc, seed coat; SE, somatic embryo. The scale bars are 2 mm in (**A**–**L**).

For the microscopy analysis, mature seed-derived calli were imaged with a Nikon SMZ1000 microscope using NIS-Elements software. Typical white and loose non-embryogenic tissue (type 2) and yellow compact embryogenic calli (type 2) can be easily distinguished by microscope (Fig. 1), “yellow or white”.

### 2.5 Histological studies

Neutral lipids in embryogenic tissue can be stained using Sudan red dye (Sudan red 7B, Yuanye, Beijing) [11,23]. Whole calli were incubated into a filtered Sudan red solution for 2 h, then rinsed 3 times with water. Images were recorded by microscopy.

Friable calli bearing somatic embryos were applied for a histological observation. Calli were fixed in an FAA solution (R20517 Yuanye, Beijing, China) for 24 h and then stored in 70% ethanol. Fixed materials were dehydrated by an absolute ethanol and xylol series, infiltrated and embedded in paraffin wax. Transverse sections with 5 μm thickness were obtained using a rotary microtome, mounted on a glass slide, and further stained with Giemsa’s stain (G8220 Solarbio, Beijing, China). Sections were observed under a Nikon 80i microscope.

### 2.6 Experimental design and statistics

The number of typical white and loose non-embryogenic tissue (type 1) and yellow compact embryogenic calli (type 2) were recorded by microscopy. 30 seeds were sowed as one technical replicate, and three technical replicates from the same seed batch are calculated for each treatment. The results from each treatment agree with other independent experiments designed with several biological replicates from different mature seed batches.

Statistics were obtained using Excel 2016. Significant differences in somatic embryogenesis efficiency were calculated by two-tailed student’s *t*-test. Error bars represent the standard deviation. n = 3 (*p < 0.05; **p < 0.01).

## 3 Results

### 3.1 2,4-D gradient induces two types of calli from plumular axis cells

Zygotic embryos from mature sorghum seeds could be readily switched from normal seedling development to a somatic embryogenesis pathway in the presence of 2,4-D [18]. The tissue culture protocol presented here is a modified version of a previous protocol [11], and uses 2,4-D as the inducer and germinating mature seeds as explants. Morphological and histological identity were investigated to describe the SE developmental process. The protocol continuously germinated and cultured Jinza-12 sorghum seeds on a solid medium with 2,4-D.

**Fig. 2:**
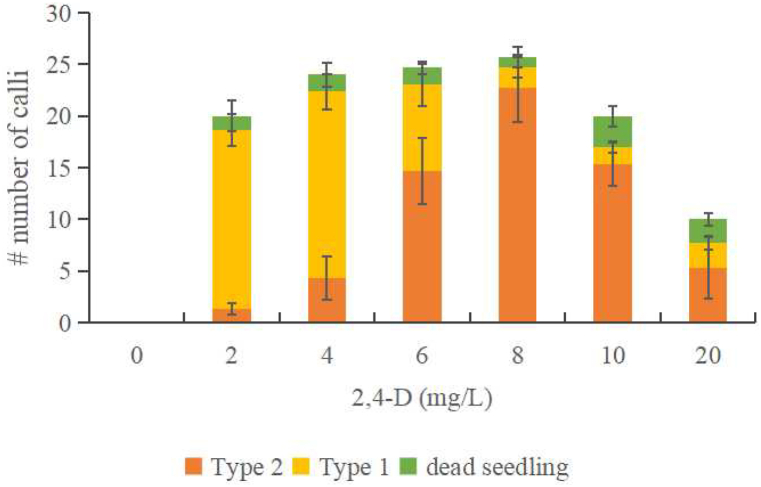
Effect of 2,4-D gradient on callus induction from mature sorghum seeds.

During the first 14 days of culture, the seedling’s plumular axis enlarged, and the epidermal and cortex cells on it began to form callus (Fig. 1A, B, G, H). By 28 days of culture, non-embryogenic (type 1) and embryogenic (type 2) calli could be distinguished morphologically: type 1 is a loose cotton-like callus induced from low concentration 2,4-D treatments, while type 2 is friable yellow embryogenic calli from high-concentration 2,4-D treatments. The type 2 callus could regenerate into an entire plantlet (Fig. 1G–L). The majority of embryogenic calli had an elongated plumular axis region, on which the somatic embryos started to detach from vascular tissue inside the calli. Globular somatic embryos were visible on calli from day 50 of culture and onward (Fig. 1J). A non-embryogenic callus (type 1) turns brown and eventually stops growing (Fig. 1C–F). We found that ca. 40–70% of seedlings produce calli depending on different batches. In general, the ratio of two types of calli are strongly in line with the 2,4-D gradient treatment (Fig. 2). The embryogenic process, with its obvious callus phase, suggests that somatic embryos developed from an indirect SE pathway [9]. In this study, we show that high concentration of 2,4-D (eg: 8 mg/L) can strongly induce SE process from mature sorghum seeds, which can be used as a fast and reliable protocol of preparing genome editing recipient in the future.

### 3.2 Time window and 2,4-D gradient of SE induction

**Fig. 3:**
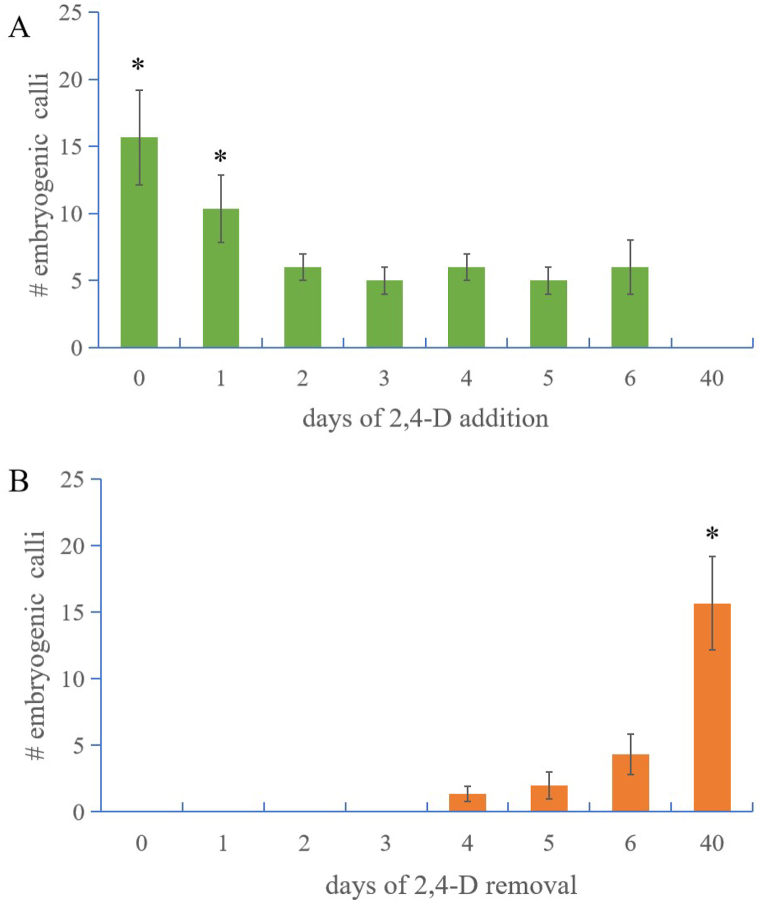
Effect of 2,4-D removal and addition on somatic embryogenesis. (**A**) 2,4-D addition. 2,4-D (8 mg/L) was added on the first day of culture (0), and each day after (1–6). (**B**) 2,4-D removal. 2,4-D was added on the first day of culture (0) and then removed at indicated time points by transferring the explant onto an MS medium without 2,4-D. A continuous 40 day 2,4-D treatment was presented as the control. Significant differences in somatic embryogenesis efficiency were calculated by a Student’s *t*-test (*P < 0.05). Error bars: SD value from three replicates of one experiment.

We further determined the time window wherein 2,4-D is necessary to stimulate SE by removing or adding 2,4-D from designed time points (Fig. 3A and B). A continuous 2,4-D treatment is a prerequisite for inducing embryogenic calli (type 2). Seedlings which are 1–6 days old can be used to induce somatic embryos. The induction rate dropped significantly from day 2 in the germination stage (Fig. 3A). Undifferentiated parts of the seedling, for example, plumular axis cells and the basal leaf sheath, have the potential for embryogenesis. The addition of 2,4-D at the start of culturing showed the highest amount of SE, while adding 2,4-D at gradually later time points was in line with a decreased SE amount, such that SE could be poorly induced when 2,4-D was added after the fourth day of culture (Fig. 3). The duration of continuous treatment of 2,4-D is necessary to induce embryogenic calli (type 2). Removal of 2,4-D from the start of culturing significantly decreased SE induction, while removal at later time points showed an increasing SE amount. Even 6 days of 2,4-D treatment is not enough to reach the highest yield of SE, and the induced calli are usually non-embryogenic (type 1), far from the calli continuously induced by the 2,4-D treatment (Fig. 3B). The results suggest that a continuous treatment at a high concentration of 2,4-D (e.g., 8 mg/L) is the key to inducing somatic embryos from mature sorghum seeds. A lower concentration or shorter treatment duration seems suitable to induce non-embryogenic calli (type 1), while embryogenic calli (type 2) can be induced from a higher 2,4-D concentration or relatively longer treatment. The germinating seeds have a two-day time window for inducing somatic embryos.

### 3.3 Sudan red-stained embryogenic tissue

**Fig. 4:**
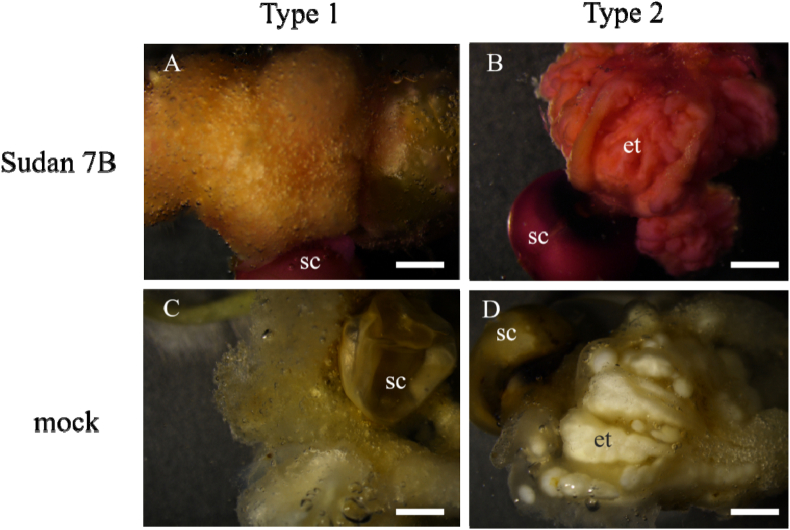
Sudan red stain-induced tissues. The non-embryogenic white and loose structure (type 1) can hardly be stained (**A**), while the yellow and compact (type 2) embryogenic tissue can be intensively stained (**B**). Type 1 (**C**) and type 2 (**D**) calli were incubated in the solvent (60% isopropanol) as a mock treatment. The pictures are light micrographs: sc, seed coat; et, embryogenic tissue. The scale bars are 2 mm.

The embryogenic tissue can be stained by Sudan red 7B. Neutral lipids, such as triacylglycerols, usually accumulate in Arabidopsis zygotes [21] and somatic embryos [8]. In the non-embryogenic calli (type 1) the tissue can be slightly stained on the surface by Sudan red (Fig. 4A), while the embryogenic calli (type 2) can be intensively stained (Fig. 4B). Type 1 (Fig. 4C) and type 2 (Fig. 4D) calli presented different qualities in the mock treatment (only 60% isopropanol).

### 3.4 Chemical enhancers improve somatic embryogenesis

**Fig. 5:**
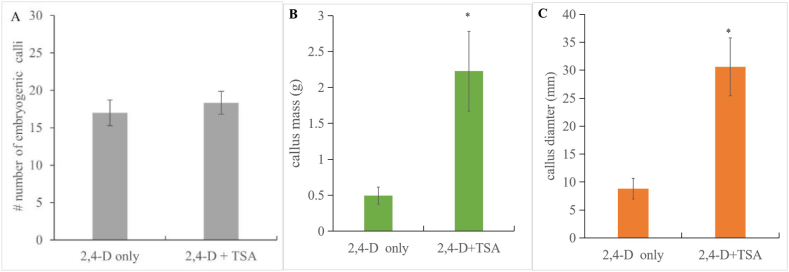
Effect of a chemical enhancer on somatic embryogenesis. The number of induced embryogenic calli (Type 2) seems not significantly improved by enhancer TSA (**A**). Importantly, the mass (**B**) and diameter (**C**) of calli were improved by 2,4-D and TSA co-treatments, measured from ten embryogenic calli, TSA treatment and only 2,4-D (8 mg/L). Significant differences were calculated by a Student’s *t*-test (*p < 0.05). Error bars: SD value from ten embryogenic calli.

Productive embryogenic calli can be observed after 2,4-D and TSA co-treatments. The efficiency of embryogenic calli (Type 2) induction seems not significantly improved (Fig. 5A). Interestingly, embryogenic calli (Type 2) from 2,4-D and TSA co-treatments show more productivity and looks more friable and flatten on the medium. TSA boosts the mass (Fig. 5B) and diameter (Fig. 5C) of induced embryogenic calli (Type 2). Technically, 8 mg/L 2,4-D combined with 0.5 μM TSA can improve the SE productivity from mature sorghum seeds, which showing bigger size and mass (Fig. 5B and C).

### 3.5 Somatic embryogenesis from different genotypes

**Fig. 6:**
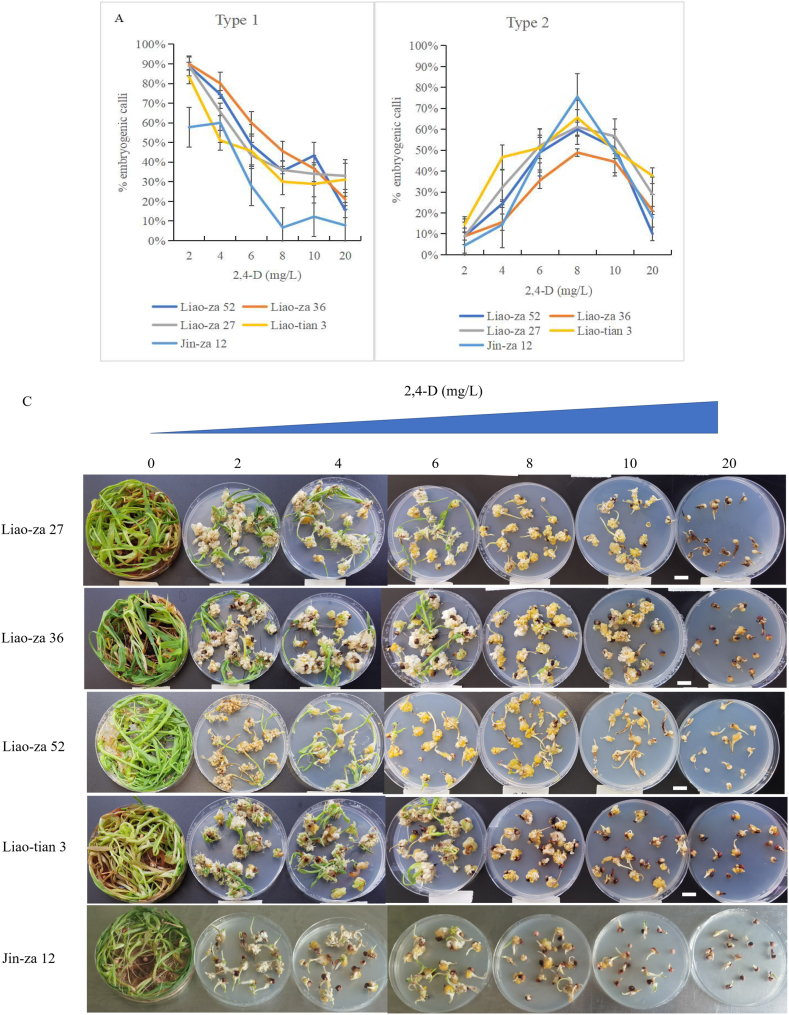

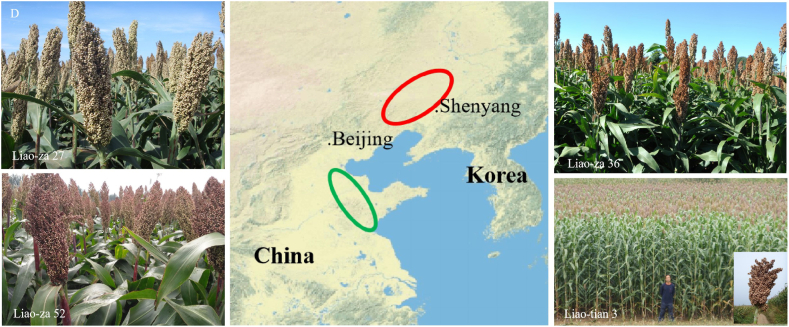
Somatic embryogenesis from different genotypes. Mature seeds of five sorghum hybrid varieties: ‘Liao-za 27’, ‘Liao-za 36’, ’Liao-za 52’,‘Liao-tian 3’ and ’Jin-za 12’ were applied for somatic embryo induction by a 2,4-D gradient. ‘Liao-za 27’, ‘Liao-za 36’, ’Liao-za 52’,‘Liao-tian 3’were breeded by Sorghum Research Institute (National Sorghum Improvement Center), Liaoning Academy of Agricultural Sciences. The induction rate of the white loose callus (type 1) significantly dropped when the 2,4-D concentration increased (**A**). Parabolic-like tendency of type 2 embryogenic callus induction (**B**). Overview of SE induction from ‘Liao-za 27’, ‘Liao-za 36’, ’Liao-za 52’,‘Liao-tian 3’ and ’Jin-za 12’. The scale bars are 1 cm. (**C**). The phenotype and distribution of the four ‘Liao’ series variety. The cultivated area of ‘Liao-za 27’, ‘Liao-za 36’, ’Liao-za 52’ were marked in red oval; while of ‘Liao-tian 3’ were marked in green oval (**D**).

Five widely planted sorghum hybrid varieties in China — ‘Liao-za No.52’, ‘Liao-za No.36’, ‘Liao-za No.27’ ‘Liao-tian No.3’ and ‘Jin-za No.12’.—were selected to investigate somatic embryogenesis from mature seeds. ‘Liao-za No.52’, ‘Liao-za No.36’, ‘Liao-za No.27’ ‘Liao-tian No.3’ were breeded by Sorghum Research Institute (National Sorghum Improvement Center), Liaoning Academy of Agricultural Sciences, which were wildly cultivated in north China (ca. 45,000 ha). Forty days after induction, the two types of callus tissue could be observed on a plumular axis section. The induction rate was calculated in line with the 2,4-D gradient. The induction rate of a white loose callus (type 1) significantly dropped when the 2,4-D concentration increased (Fig. 6A). On the other hand, all candidates exhibited a similar parabolic tendency of type 2 callus induction (Fig. 6B). ‘Jin-za No.12’ showed the highest somatic embryogenesis rate (type 2), reaching 73%. Somatic embryogenesis did not show obvious genotype dependency in candidates (Fig. 6C). The phenotype of the four varieties (‘Liao-za No.52’, ‘Liao-za No.36’, ‘Liao-za No.27’ ‘Liao-tian No.3’) was presented in Fig. 6D). The cultivated area of ‘Liao-za 27’, ‘Liao-za 36’, ‘Liao-za 52’ were marked in red oval, while of ‘Liao-tian 3’ were marked in green oval. (Fig. 6D middle).

### 3.6 Histology of somatic embryogenic clump

**Fig. 7:**
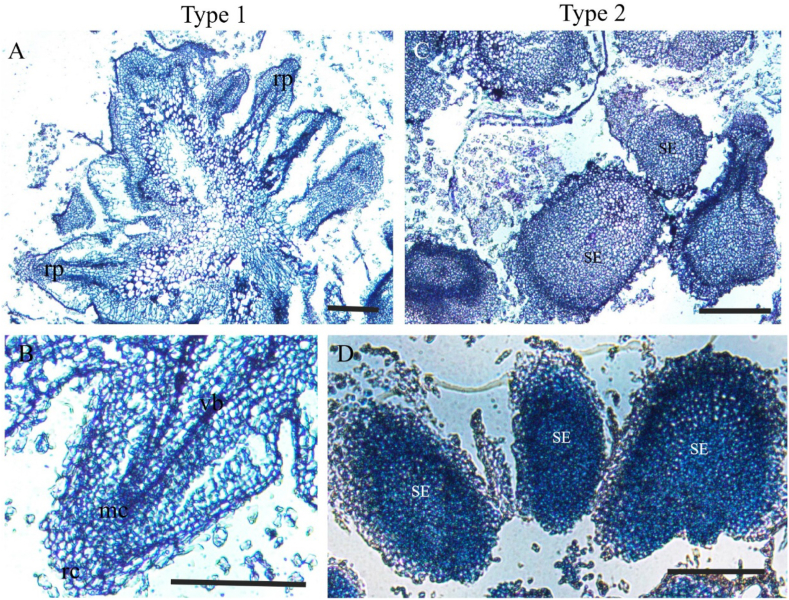
Histology of somatic embryogenesis from mature seeds in sorghum. (**A**, **B**) Type 1 white and loose non-embryogenic callus; root organogenesis from parenchyma tissues. Somatic embryogenesis 60 days after induction presenting with a low concentration of 2,4-D. (**C**, **D**) Type 2 yellow and compact embryogenic callus; somatic embryogenesis from parenchyma tissues; 60 days after callus inoculation: rp, root primordia; rc, root cap cell; mc, meristematic cells; vb, vascular bundle; SE, somatic embryo. The scale bars are 1 mm.

The inner structure of type 1 and type 2 calli was further identified histologically. Tissue sections were taken from 8-week-old calli. A transverse section of a type 1 callus (Fig. 7A) showed root organogenesis from inside the parenchymatic tissue. Meristematic cells differed from surrounding parenchyma cells by an intensively stained cytoplasm and obvious vascular elements (Fig. 7C). Globular stages of somatic embryos can be clearly found (Fig. 7B, D). SE development does not seem to be synchronized because globular and pear-shaped stages were simultaneously present in the one slide (Fig. 7D). Histological studies of calli indicate root organogenesis from type 1 and somatic embryogenesis from type 2.

## 4 Discussion

Embryo development in angiosperms can be generally divided into three phases: cell proliferation, morphogenesis and maturation [24,25]. The zygotic embryo explants utilized for regeneration are usually from the maturation phase, starting with a transition from cell division to expansion; a large amount of storage products are accumulated thereafter [22]. Immature zygotic embryos (IZE) are usually applied for sorghum somatic embryo induction [15,26], showing the highest competence when continuously treated with 2,4-D [10]. Nevertheless, somatic embryogenesis from mature seeds in sorghum has hardly been investigated [18]. IZE and mature seed explants are both from the seed maturation phase and did not show a significant difference in somatic embryogenesis. SE induced from mature seeds has been reported in many crops, such as Arabidopsis [11], rice [20], wheat [27], maize [28], peanut [29], magnolia [30] and grape [28]. On the other hand, histone deacetylation inhibitor TSA can enhance microspore embryogenesis [31]. Decreased global DNA methylation by 5-azacytidine was also found to promote embryogenesis combined with 2,4-D [29]. The network of hormone transcription factors modulating SE restricted by chromatin-modifying proteins has been well investigated in Arabidopsis [9], but it has hardly been studied and utilized in crops.

In this study, we show that a relatively high concentration of 2,4-D (eg: 8 mg/L) can efficiently induce somatic embryogenesis from mature sorghum seeds, whose SE productivity can be boosted by deacetylation inhibitor (Fig. 5). Our finding provides a novel and handy SE system from mature sorghum seeds, which can also accelerate recipient preparation of genome editing in the future.

### 4.1 High-concentration 2,4-D induces somatic embryo clusters from mature sorghum seeds

Exogenous 2,4-D can induce two types of calli in Poaceae [20,21,32]. In these systems, mature seeds are cultured in a certain concentration of 2,4-D to induce the white non-embryogenic calli (type 1) and yellow friable embryogenic calli (type 2) [20], which shares similar SEs structure and developmental progress to our results (Fig. 1). The regeneration efficiency varies among different genotypes in rice [30,33]. When building regeneration system for new varieties, different culture media such as 2N6 and 2NBK were highly recommended to be tried for different genotypes [33]. Here, SE is investigated from a wide 2,4-D gradient treatment, and yellow friable embryogenic calli (type 2) can be found directly from the plumular axis cells when treated with a higher concentration of 2,4-D (Fig. 1) Embryogenic calli (Type 2) seem longer in maize [32], and loose in rice [34]. The non-embryogenic calli (type 1) can be frequently observed from low concentration 2,4-D treatments, which are white and loose (Fig. 1). Type 2 protuberances are initiated from plumular axis cortex cells 20 days after culturing (Fig. 1). Technically, 6∼10 mg/L 2,4-D can efficiently induce somatic embryogenesis from mature sorghum seeds. Our efficient SE protocol does not show obvious genotype dependency in the five candidates (Fig. 6A and B), which is a potential genome editing recipient system.

The tissue competence for somatic embryogenesis differs within certain explants. For example, SE can develop from either the shoot apex [11] or cotyledon petioles [35] from an Arabidopsis embryo culture system. According to the different SE performance of ABA and auxin signaling mutants, two molecular mechanisms seem to be in charge of SE originating tissue from seedling explants [8]. Somatic embryogenesis from ABA biosynthesis mutants (e.g., *aba2-1*) and positive ABA signaling mutants (e.g., *snrk2*) show a reduced competence for somatic embryogenesis from the SAM region, but increased callus formation in bipolar regions [8]. In sorghum, SEs can be induced from the nodal region of the germinating seedling isolated SAM section, where the subtending node can produce SE calli in the presence of 2.5 mg/L 2,4-D [18], which is different from our finding (Fig. 1, Fig. 4) that SE is directly developed from plumular axis cortex cells. This difference in tissue competence seems to be due to the relative expression level of embryogenic transcription factors [8,36], such as LEC1, ABI3 and FUS3, and genotype dependency. Overall, we have shown that the SE rate from mature seeds is around 40–70%, which is in line with previous studies that observed 2,4-D-induced somatic embryogenesis during the process of seed development and post-harvest ripening [11,37]. In brief, there is a para-curve-like tendency of 2,4-D gradient-induced embryogenic calli (type 2) from mature sorghum seeds (Fig. 6B). SE efficiency is significantly determined by the 2,4-D concentration, whereby highlights the convenience of recipient preparation in the future.

### 4.2 Developmental window of somatic embryogenesis

Hormone transcription factors inducing somatic embryogenesis, such as LAFL and ABI [9], were found to converge on the auxin and ABA signaling pathway within a narrow developmental window [8]. ABI and LAFL genes seem be regulated by larger complex transcriptional feedback loops during seed mutation [37]. The 2,4-D addition and removal experiment indicated that there is a wider developmental window in sorghum than Arabidopsis, wherein the foreign 2,4-D treatment is the most effective (Fig. 3) [8]. 2,4-D uptake is usually ensured by influx transporters but they may be hardly secreted out by efflux transporters [38], which make 2,4-D continuously function for a longer duration and show poor mobility after absorption. Seedlings are composed of various cell types, and only a subset of the plumular axis contributes to somatic embryogenesis competence. Arabidopsis seeds are small and have a narrow developmental window of embryogenic competence [8]; meanwhile, sorghum seeds are bigger and contain more competent cells, which can produce SE and show a wider time window than that of Arabidopsis (Fig. 1, Fig. 3).

Additional studies on mutant analyses [8] and pharmacological intervention [39,40], as well as omics data [9], will further resolve the detailed contributions of different hormone signals within this developmental window. Furthermore, it would be more convincing to study the expression pattern of some SE transcription factors, such as BBM or WUS, on transcriptome and proteome level within the time window.

### 4.3 Chemical enhancer TSA improves SE productivity

The molecular mechanism of somatic embryogenesis has been deeply investigated in the past decades [31]. Morphogenic transcription factors, also known as developmental regulators (DRs), manipulate somatic embryogenesis. For example, overexpression of BBM, WUS [31], Wound Induced Dedifferentiation (WIND) [31] can trigger somatic embryogenesis or callus formation. Some of them were successfully used in sorghum transformation [31]. Interestingly, the network of morphogenic transcription factors among hormone signaling pathway can be regulated on epigenetic level [31]. Trichostatin A (TSA) is highly effective at a late stage of microspore development, and shows a strong effect on sporophytically dividing cells [31]. TSA usually binds to the zinc-containing motifs of HDACs and then performs its biological function [40]. Epigenetics seem to manipulate the repression effect on the cell cycle progress, which is slowly imposed on competent tissues; the gradual release of this repression seems necessary to somatic embryogenesis [9,31]. A microarray analysis indicates that TSA can also induce the expression of genes involved in the G1/S cell cycle and initiated embryogenesis [31]. Technically, 6∼10 mg/L 2,4-D combined with 0.5 μM TSA can significantly improve the SE productivity from mature sorghum seeds, which is useful to build genome editing recipient system.

Cultured explants may regulate their epigenetic program to ultimately adapt the artificial hormonal environment [41].Our microscopic examination suggests that massive embryogenic tissues can be induced by exogenous TSA treatment (Fig. 5), which is in line with previous reports. Interestingly, TSA treatment is also involved in the upregulated expression of cell wall mobilization enzymes, especially those associated with the mobilization of pectin [31]. Our results indicate that in vitro germinated SE can be enhanced by TSA, which is also noted on pectin-rich surface mucilage. This mucilage can also be found in *Brassica napus* [42], *Cocos nucifera* [43] and *Acrocomia aculeata* [44] when cultured in vitro. Mucilage on the surface increases surface contact, thereby allowing for better nutrient absorption from the medium. The presence of mucilage in explants may be a strong indicator of successful somatic embryogenesis [44].

Here we show that some deacetylation inhibitor can strongly improve SE productivity in Sorghum (Fig. 5). Nevertheless, the molecular mechanism improving SE productivity between auxin signaling pathway and epigenetics level still need to be presented in the future study.

### 4.4 Histology of the sorghum embryogenic clump

Densely stained SE can be observed on the surface of calli 8 weeks after induction. Similar descriptions of embryogenic cells have previously been reported [42]. In Poaceae, somatic embryos usually went through a globular stage, a pear-shaped stage, a mature embryo stage and finally regenerated into a plantlet [45]. Rapidly dividing cells on the epidermis of proliferating calli give rise to somatic embryos. Embryogenic identity was acquired in these proembryos, which resulted in the formation of globular and pear-shaped stage SE (Fig. 7B, D). Newly formed somatic embryos have a dense protoplasm, which can be intensively stained. In the present observation, some somatic embryos were fused to each other (Fig. 4B, D), due to cell division in the meristem prior to the differentiation of shoots and cotyledons [46].

## 5 Conclusions

Somatic embryos can be induced from mature sorghum seeds by a high concentration of 2,4-D. When 2,4-D is combined with certain deacetylation inhibitor, the productivity of SE is improved.

## Funding

This research was funded by Fellowship of China Post-doctoral Science Foundation (2021M693846); National Millet Sorghum Industrial Technology System Sorghum Cultivation Station Scientist (CARS-06-14.5-A22) and Forage Sorghum Breeding Station Scientist (CARS-06-14.5-A11); 10.13039/501100012166National Key R&D Program of China, “screening of high-quality, special, high-yield and high-efficiency varieties for cereals” (2019YFD1001704); General Project of the Dean's Fund of 10.13039/501100020199Liaoning Academy of Agricultural Sciences, China (2021MS0504); Liaoning Province “Storing Grain in Technology” Major Special Project (2023JH1/10200001); Creation and Utilization of High-Quality Germplasm of Northern Glutinous Sorghum (23-410-2-15); Seed Industry Innovation Special Project in Shenyang City (22-318-2-15).

## Data availability

Sharing research data helps other researchers evaluate your findings, build on your work and to increase trust in your article. We encourage all our authors to make as much of their data publicly available as reasonably possible. Please note that your response to the following questions regarding the public data availability and the reasons for potentially not making data available will be available alongside your article upon publication. Response: No.

Please select why. Please note that this statement will be available alongside your article upon publication. As follow-up to “Data Availability Sharing research data helps other researchers evaluate your findings, build on your work and to increase trust in your article. We encourage all our authors to make as much of their data publicly available as reasonably possible. Please note that your response to the following questions regarding the public data availability and the reasons for potentially not making data available will be available alongside your article upon publication. Response: Data included in article/supp. material/referenced in article.

## CRediT authorship contribution statement

**Han Wu:** Writing – original draft, Supervision, Project administration, Methodology, Funding acquisition. **Kuangye Zhang:** Software, Methodology, Conceptualization. **Jia Li:** Methodology. **Jiaxu Wang:** Methodology. **Yanqiu Wang:** Methodology. **Junchi Yu:** Methodology. **Ling Cong:** Methodology, Formal analysis, Data curation. **Youhou Duan:** Resources. **Fulai Ke:** Formal analysis, Data curation. **Fei Zhang:** Resources, Methodology. **Zhiqiang Liu:** Resources. **Feng Lu:** Formal analysis, Data curation. **Zhipeng Zhang:** Writing – original draft, Funding acquisition, Conceptualization. **Jianqiu Zou:** Supervision, Funding acquisition, Conceptualization. **Kai Zhu:** Validation, Supervision, Resources.

## Declaration of competing interest

The authors declare the following financial interests/personal relationships which may be considered as potential competing interests:Han Wu reports financial support was provided by Fellowship of China Post-doctoral Science Foundation (2021M693846). If there are other authors, they declare that they have no known competing financial interests or personal relationships that could have appeared to influence the work reported in this paper.
